# Impact of Selection for Digestive Efficiency on Microbiota Composition in the Chicken

**DOI:** 10.1371/journal.pone.0135488

**Published:** 2015-08-12

**Authors:** Sandrine Mignon-Grasteau, Agnès Narcy, Nicole Rideau, Céline Chantry-Darmon, Marie-Yvonne Boscher, Nadine Sellier, Marie Chabault, Barbara Konsak-Ilievski, Elisabeth Le Bihan-Duval, Irène Gabriel

**Affiliations:** 1 UR83 Recherches Avicoles, INRA, Nouzilly, France; 2 INRA, LABOGENA, Domaine de Vilvert, Jouy en Josas cedex, France; 3 INRA, UE1295 PEAT, Nouzilly, France; Max Rubner-Institut, GERMANY

## Abstract

**Objectives:**

Feed efficiency and its digestive component, digestive efficiency, are key factors in the environmental impact and economic output of poultry production. The interaction between the host and intestinal microbiota has a crucial role in the determination of the ability of the bird to digest its food and to the birds’ feed efficiency. We therefore investigated the phenotypic and genetic relationships between birds’ efficiency and the composition of the cecal microbiota in a F2 cross between broiler lines divergently selected for their high or low digestive efficiency.

**Methods:**

Analyses were performed on 144 birds with extreme feed efficiency values at 3 weeks, with feed conversion values of 1.41±0.05 and 2.02±0.04 in the efficient and non-efficient groups, respectively. The total numbers of *Lactobacillus*, *L*. *salivarius*, *L*. *crispatus*, *C*. *coccoides*, *C*. *leptum* and *E*. *coli* per gram of cecal content were measured.

**Results:**

The two groups mainly differed in larger counts of Lactobacillus, *L*. *salivarius* and *E*. *coli* in less efficient birds. The equilibrium between bacterial groups was also affected, efficient birds showing higher *C*. *leptum*, *C*. *coccoides* and *L*. *salivarius* to *E*. *coli* ratios. The heritability of the composition of microbiota was also estimated and *L*. *crispatus*, *C*. *leptum*, and *C*. *coccoides* to *E*. *coli* ratios were moderately but significantly heritable (0.16 to 0.24). The coefficient of fecal digestive use of dry matter was genetically and positively correlated with *L*. *crispatus*, *C*. *leptum*, *C*. *coccoides* (0.50 to 0.76) and negatively with *E*. *coli* (-0.66). Lipid digestibility was negatively correlated with *E*. *coli* (-0.64), and AMEn positively correlated with *C*. *coccoides* and with the *C*. *coccoides* to Lactobacillus ratio (0.48 to 0.64). We also detected 14 Quantitative Trait Loci (QTL) for microbiota on the host genome, mostly on *C*. *leptum* and Lactobacillus. The QTL for *C*. *leptum* on GGA6 was close to genome-wide significance. This region mainly includes genes involved in anti-inflammatory responses and in the motility of the gastrointestinal tract.

## Introduction

Feed efficiency is the major component of both economic profitability and environmental impact of poultry production. It has been shown that when birds are fed a challenging diet (for their hardness and viscosity characteristics) their digestive efficiency has a significant role in feed efficiency, and that is highly heritable [1]. After 8 generations of divergent selection on digestive efficiency, we obtained two genotypes of chickens with 30 to 40% difference between good digesters (D+) and poor digesters (D-). In a preliminary study, Gabriel et al. [2] showed that the composition and homogeneity of microbiota varied widely between these two lines, which suggests that the genetics of the host influence the composition of its microbiota. This difference in microbiota composition is not surprising as biotopes of the digestive tract have probably been modified between these two lines due to differences in anatomy of the gastrointestinal tract and digestive physiology [3]. Moreover, the microbiota is in constant interaction with the host and has been shown to influence several major functions such as the immunological, physiological and nutritional status of birds [4,5]. Several factors originating from the host can impact its microbiota, such as those due to digestive physiology (turnover of the intestinal epithelium, quantity of mucus, motility of gut, gut secretions), the nutrient composition of the bowel which depends on the composition of the diet and on the bird’s capacity to digest feed, and the presence of antibacterial compounds of the immune system [6].

Several studies have suggested the existence of the influence of the host’s genetics on the composition of the chicken microbiota as it differs between individuals [7,8], between lines selected on growth or digestive efficiency [2,4,9–12] and between birds within a genotype with high or low feed or digestive efficiency [9,11,13]. A few studies have gone further than group comparisons, to propose estimates of genetic parameters, genetic or phenotypic correlations between growth performance and microbiota composition or QTL detection for microbiota. Heritability of the quantity of 16S rRNA copies has been estimated in only two studies on high and low body weight chicken lines [4,10]. Despite the relatively low numbers of birds (60 to 132 chickens per study), they indicated that some species or genera such as Lactobacillus spp. or Streptococcae seem to be heritable and correlated with body weight. However, no correlation with feed or digestive efficiency was available, and these studies relied on fecal samples, the composition of which varies widely within a day due to emptying of the ceca [14]. However, studies performed in mammals have indicated that the host’s genetics influence its digestive microbiota [15].

The aim of our study was therefore to provide a complete set of information on the genetic basis of the host’s influence on microbiota composition through i) comparison of microbiota composition between high and low efficiency groups to establish how far selection on digestive efficiency affected microbiota composition, ii) estimation of phenotypic and genetic relationships between feed efficiency, digestive efficiency and microbiota composition to estimate which proportion of microbiota composition is due to the overall genetic background of birds and iii) Quantitative Trait Loci (QTL) detection of microbiota composition to identify regions in which a variation of the DNA sequence will affect microbiota composition, through a modification of the environment provided to bacteria (e.g., physico-chemical conditions, nutrient concentrations, immune system activity, …). As previous studies showed that the difference in microbiota composition between efficient and non-efficient birds was greater in cecal content than in other intestinal compartments [2,11,16], we focused our study on this specific digestive segment.

## Materials and Methods

### Animals and rearing

All animal care and experimental procedures reported in this paper were in accordance with French and European regulations concerning animal experimentation, including authorizations to experiment on live birds no. 37–100, 006290, 37–123, 37–005, A37-162, 04726, 7275 for scientists and those delivered at 30/09/1996, 26/02/2007, and 09/09/2005 for technicians from the French Ministry of Agriculture. The Experimental Unit where birds were kept is registered by the ministry of Agriculture with license number C-37-175-1 for animal experimentation. Measure of digestive efficiency in individual cages, blood sampling procedures for genotyping, euthanasia procedures by injection of pentobarbital, scientific justification, evidence for a lack of alternatives and endpoints were approved by the ethics committee in Animal Experimentation of Val de Loire (00886.02 and 01047.02). This ethics committee is registered by the National Committee under the number C2EA-19. The personal license number from the French Veterinary Service for this study is 548.

Data were collected on chickens from a F2 population obtained by crossing two medium-growth broiler lines divergently selected on their high (D+) or low (D-) digestive efficiency determined by metabolizable energy corrected to zero nitrogen retention at 3 weeks (AMEn) [1]. The divergent selection experiment started from a pure male line from the SASSO breeding company, used as the father of the medium-growth crossbred commercial chickens. The F2 population, created to detect the QTLs for digestive efficiency, has been described in Tran et al. [17]. Before crossing, the D+ and D- populations presented wide differences in feed and digestive efficiency (30 to 40%) [18,19]. Using an F2 population instead of the initial divergent lines allowed us to compare efficient and non-efficient birds in a population with a common genetic background.

Five males and fourteen females per line (D+ and D-) were used as F0 grand-parents. Males of the D+ and D- lines were mated respectively to females from the D- and D+ line to produce the F1 generation (half D+×D- and half D-×D+). Six F1 sires (3 D+×D- and 3 D-×D+) were mated to sixty F1 females of the reciprocal cross (i.e., D+×D- females for D-×D+ males, D-×D+ females for D+×D- males) to produce a total of 864 F2 birds (male and female). The F2 birds were reared in 4 batches (between January and June 2009) on the floor from hatching to 8 d to allow normal development of intestinal microbiota and subsequently transferred to individual cages in three rearing cells until slaughter at 23 d to measure feed and digestive efficiency. Birds were fed a diet similar to the diet used during the selection experiment [17], including 52.5% Rialto wheat and 6% soybean oil, 3110 kcal.kg^-1^ DM and 21.1% CP. Clinacox (0.02%) was used as anticoccidial agent, as it has a limited effect on the development of intestinal microbiota [20,21].

### Phenotypes

Birds were weighed at 0, 9, 14, 17, 20 and 23 d. Their feed intake was individually recorded between 9 and 14 d, 14 and 17 d, 17 and 20 d and between 20 and 23 d. A balance trial with a total collection of excreta was performed between 20 and 23 d to measure fecal digestive efficiency traits as AMEn, coefficients of fecal digestive use of dry matter, starch, lipids and proteins (CDUDM, CDUS, CDUL, CDUP). These digestive efficiency traits were determined through near infrared spectroscopy following the method of Bastianelli et al. [22].

Due to time constraints, it was not possible to select birds used for microbiota study on their digestive efficiency. Instead, the subsample of 144 birds used for microbiota determination were selected on their feed efficiency between 17 and 20 d, as feed efficiency and digestive efficiency had been previously shown to be strongly genetically correlated (-0.70, [1]). The mean values of feed efficiency, estimated through the feed conversion ratio (FCR, i.e. the ratio of feed intake to weight gain) were 1.41±0.05 in the low FCR group (FCR_L) and 2.02±0.04 in the high FCR group (FCR_H), respectively. Initial and final body weight of birds in the 2 groups were similar (Table 1), as could be expected from the absence of genetic correlation between AMEn and body weight.

**Table 1:** Least square means (± standard errors) of feed and digestive efficiency, body weight and feed intake in the high (FCR_H) and poor (FCR_L) feed efficiency groups.

Traits^1^	FCR_L	FCR_H	Significance of FCR group effect
**FCR (g.g** ^**-1**^ **)**	1.41 ± 0.05	2.02 ± 0.04	<0.0001
**AMEn (kcal.kg** ^**-1**^ **DM)**	3444 ± 44	2936 ± 39	<0.0001
**CDUDM (%)**	73.1 ± 0.9	63.1 ± 0.8	<0.0001
**CDUS (%)**	98.4 ± 1.1	88.2 ± 0.9	<0.0001
**CDUL (%)**	84.8 ± 2.0	61.6 ± 1.8	<0.0001
**CDUP (%)**	84.8 ± 0.6	76.8 ± 0.6	<0.0001
**BW20**	327.1 ± 6.0	319.5 ± 6.9	0.35
**BW23**	426.3 ± 7.8	427.2 ± 8.92	0.92
**FI**	212.7 ± 4.1	166.5 ± 4.6	<0.0001

^1^ FCR: feed conversion ratio between 17 and 20 d; AMEn: metabolisable energy corrected to zero nitrogen retention between 17 and 20 d; CDUDM, CDUS, CDUL, CDUP: coefficients of fecal digestive use of dry matter, starch, lipids, and proteins between 17 and 20 d; BW20, BW23: body weight at 20 and 23 days; FI: feed intake between 20 and 23 days.

### Microbiota determination

Previous studies in these chicken lines showed that the greatest difference in microbiota composition between D+ and D- was in the cecal content [2]. This study was therefore focused on this digestive segment. At 23 d, after 2h30 of feeding following 8 hours’ fasting, birds were killed by pentobarbital injection, and their ceca immediately removed. Ceca were opened and their content gently removed in order to obtain only the content and not the mucosa, frozen in liquid nitrogen and stored at -80°C until further processing.

Microbial DNA was then extracted from cecal samples using the QIAamp DNA mini-kit (QIAGEN, cat#51306). We used a combination of the methods of Yu et al. [23] and Stanley et al. [11]. Briefly, 25 mg of cecal content were transferred to a tube with lysis buffer [11] and sterile zirconium beads. Samples were homogenized at maximum speed (Frequency 30.sec^-1^) Retsch MM301 for 3 min, followed by heating at 70°C for 5 min. Following centrifugation (5 min, 16 000 g, 4°C), a second extraction step was carried out. The two supernatants were pooled for the DNA purification step. Proteinase K was added and the sample was heated at 70°C for 10 min to remove proteins. Ethanol was then added and the sample was purified using a QIAamp column as described by the manufacturer. The sample was eluted in Tris-EDTA buffer AE (Qiagen). DNA quantity and quality were measured on a Nanodrop spectrophotometer.

16S rDNA was quantified by qPCR to determine the number of copies of the main bacterial groups in the chicken gut within the Firmicutes phylum (lactobacillus genus, *Lactobacillus salivarius* and *Lactobacillus crispatus* species, *Clostridium coccoides* and *Clostridium leptum* groups) and within the Proteobacteria phylum (*Escherichia coli*). The primers used for Lactobacillus were those described by Walter et al. [24] and Heilig et al. [25] for forward and reverse primers, respectively. The primers used for *L*. *crispatus*, *L*. *salivarius*, *C*. *leptum*, *C*. *coccoides* and *E*. *coli* were those proposed by De Backer et al. [26], Harrow et al. [27], Matsuki et al. [28], Matsuki et al. [29] and Huijsdens et al. [30], respectively.

Reactions were run in triplicate in 384 well plates in a final volume of 10 μl. The EpMotion 5070 liquid handling robot (Eppendorf, Le Pecq, France) was used to distribute the master mix and DNA to the 384 well plates. The *L*. *salivarius* reaction consisted of 5 μl of TaqMan Universal PCR 2 × Master Mix (Applied Biosystems, Courtaboeuf, France), 0.2 μl of both 10 μM primers (Eurogentec, Angers, France) and minor groove binder probe (Applied Biosystems), 1.9 μl of nuclease-free water and 2.5 μl of template DNA at the appropriate dilution. Amplification was carried out with a Light Cycler 480 (Roche, Meylan, France) as follows: 10 min at 95°C, followed by 45 cycles of denaturation (10 s at 95°C), annealing (30 s at 60°C) and extension (30 s at 72°C). Reactions for the other bacterial groups consisted of 5 μl of Light Cycler 480 SYBR Green I Master Mix (Roche, Meylan, France), 0.5 μl of 10 μM primers (Eurogentec), 1.5 μl of nuclease-free water and 2.5 μl of template DNA at the appropriate dilution. The cycling conditions were as follows: 10 min at 95°C, then 45 cycles of denaturation (10 s at 95°C), annealing (20 s at 60°C) and extension (30 s at 72°C). Following amplification, melting curve analysis was included in order to assess the specificity of the amplified product. Standard curves were generated by amplification of serial 10-fold dilutions of *E*. *coli* (K12-1 strain, CIRM-BP 371), *L*. *plantarum* (DSM 20174, ATCC 14917, CIRM-BIA 466) genomic DNA (International Center of Microbial Resources, CIRM, INRA, France), *L*. *Salivarus* (ATTC 11741, DSMZ 20555) and *L*. *crispatus* (ATCC 33820, DSMZ 20584) and used for the quantification of total bacteria and *E*. *coli* and lactobacillus spp. *L*. *salivarus* and *L*. *crispatus*, respectively. Reference clones EF445158 and EF445150 [31] were used to generate the standard curves for the quantification of *C*. *leptum* and *C*. *coccoides*, respectively. The copy number for each reaction was calculated from the standard curves and determined by the second derivative maximum method [32]. Results are presented as number of 16S rDNA copies expressed per gram of fresh sample (Table 2).

**Table 2:** Least square means (± standard errors) of cecal microbiota counts (qPCR) in group with low (FCR_L) or high (FCR_H) feed conversion ratio.

Bacteria	FCR_L	FCR_H	Significance of group effect
**Bacterial count (log10 number of copies per g of cecal content)**			
**Lactobacillus**	11.17±0.07	11.39±0.06	0.0060
***L*. *salivarius***	10.50±0.12	10.96±0.10	0.0011
***L*. *crispatus***	11.24±0.10	11.33±0.09	0.4804
***C*. *leptum***	10.97±0.05	10.89±0.04	0.1343
***E*. *coli***	9.73±0.13	10.14±0.11	0.0071
***C*. *coccoides***	10.96±0.04	10.94±0.04	0.8136
**Ratios of bacterial count (log10 number of copies per g of cecal content) of different bacteria categories**			
***L*. *salivarius*/Lactobacillus**	0.941±0.009	0.963±0.007	0.0327
***L*. *salivarius*/*L*. *crispatus***	0.935±0.011	0.970±0.010	0.0085
***L*. *salivarius*/*C*. *coccoides***	0.958±0.011	0.993±0.010	0.0087
***L*. *salivarius*/*C*. *leptum***	0.958±0.011	0.998±0.010	0.0034
***L*. *salivarius*/*E*. *coli***	1.087±0.015	1.087±0.013	0.9536
***L*. *crispatus*/Lactobacillus**	1.007±0.005	0.995±0.005	0.0571
***L*. *crispatus*/*C*. *coccoides***	1.028±0.009	1.029±0.009	0.8715
***L*. *crispatus*/*C*. *leptum***	1.027±0.010	1.034±0.010	0.4916
***L*. *crispatus*/*E*. *coli***	1.161±0.018	1.122±0.016	0.0689
***C*. *leptum*/Lactobacillus**	0.983±0.007	0.961±0.006	0.0062
***C*. *leptum*/*C*. *coccoides***	1.317±0.115	1.105±0.108	0.1297
***C*. *leptum*/*E*. *coli***	1.138±0.016	1.084±0.015	0.0065
***C*. *coccoides*/Lactobacillus**	0.982±0.006	0.966±0.005	0.0199
***C*. *coccoides*/*E*. *coli***	1.138±0.015	1.087±0.014	0.0076
***E*. *coli*/Lactobacillus**	0.873±0.011	0.892±0.010	0.1702

As preliminary studies showed that the Bacteroides genus was not detected in F0 birds from D+ and D- lines [2], this group was not included in the present study.

### Markers and genotyping

All F0, F1 and F2 birds were genotyped with a dedicated Illumina Infinium custom array including 6,000 single nucleotide markers (SNP) markers chosen for their informativity in our design and for their distribution across the genome [33]. The markers presenting deviations from the Hardy-Weinberg equilibrium within families, inconsistent genotyping relative to pedigree or genetic map information or poor quality of markers were discarded from the analysis in order to reduce the risk of erroneous results [33]. Finally, 3,379 markers were used. The genetic map was deduced from the physical position of the SNP markers and from the genetic consensus reference map published by Groenen et al. [34]. This set of markers covers 3,099.1 cM.

### Statistical analyses

#### Phenotypic analyses

We first tested whether the composition of microbiota (count of each category or ratio of counts between categories) and digestive efficiency parameters were different between the feed efficiency groups. Due to their non-normal distributions, raw bacterial counts were log-transformed before analysis and the ratios calculated with the log-transformed counts of each category. The analysis of variance was performed with the GLM procedure of SAS/STAT Version 9.4 and model 1:

$$y_{ijklmn}=\mu +H_{i}+S_{j}+C_{k}+RC_{l}+G_{m}+e_{ijklmn}$$ (1)

where y_ijklmn_ is the performance of animal n (N = 144), μ the general mean, H_i_ the fixed effect of hatch i (i = 1 to 5), S_j_ the fixed effect of sex j (j = males or females), C_k_ the fixed effect of rearing cell k (N = 3), RC_l_ the fixed effect of the raw of cage within the cell (N = 3), G_m_ the fixed effect of FCR group (FCR_H, FCR_L), and e_ijklmn_ the residual pertaining to animal n.

In order to determine which microbiota characteristics might be related to digestive efficiency, we carried out multifactorial correspondence analysis with the SPAD 7.0 software. For each trait of microbiota and digestive efficiency, we determined three categories with equal frequencies, i.e. the third with the lowest values, the third with the median values and the third with the highest values (noted L, M and H, respectively). The analysis was performed using microbiota as active traits (i.e. contributing to construction of axes) and projecting digestive efficiency categories on the graph. The impact of active variables was assessed through their relative contribution to each axis. A t-test was then performed to determine whether digestive efficiency traits were significantly associated with composition of microbiota.

#### Genetic analyses

In order to estimate both heritability of microbiota traits and their genetic correlations with digestive efficiency, we added an animal genetic effect into the model used for the analysis of variance (model 1) and estimated genetic parameters with VCE 6.0.2 software [35]. The pedigree used to construct the relationship matrix included 1571 animals.

#### QTL analysis

QTL detection was carried out with the QTLMap software [36] using a half-sib model [37,38] with interval mapping based on maximum likelihood estimations [39]. This model does not make assumptions on the number of QTL alleles segregating in the design. The traits were analyzed separately. Depending on preliminary analysis of variance, data were pre-corrected for fixed effects of batch (4 levels), sex (2 levels), rearing cell (3 levels) and cage row (3 levels). QTL analyses were performed by comparing the hypothesis of one QTL (H1) versus no QTL (H0) to test the segregation of a QTL on each linkage group. For chromosome Z, separate analyses were performed for males and females.

For each trait on each chromosome, the significance threshold at the chromosome-wide level was calculated from the results of 5,000 simulations of performance under the null hypothesis. For the most significant QTLs, 20,000 simulations were made to derive the genome-wide *p*-value (P_G_) from the chromosome-wide *p*-value (P_C_) using an approximate Bonferroni correction:

$$P_{G}=1-{\left(1-P_{C}\right)}^{\frac{1}{r}}$$ (2)

where r is the ratio of the length of a specific chromosome to the length of the genome considered for QTL detection, as in Tilquin et *al*. [40]. Confidence intervals for QTLs (95%) were estimated using the LOD drop-off method as proposed by Lander and Botstein [39].

The significance of the QTL effects within each sire family was tested using a Student test, by assuming an equal distribution of the QTL alleles in the progeny. A QTL effect was retained as significant for Student test *p*-values <0.05, and the corresponding sire families were assumed to segregate for this QTL. These familial substitution effects were estimated in families found to significantly segregate for the QTL.

## Results and Discussion

### Differences between groups and multifactorial analysis

The main differences in digestive and feed efficiency and in microbiota composition between the FCR_L and FCR_H groups are presented in Tables 1 and 2, respectively. Differences in FCR between the two groups were due to higher feed intake in FCR_H, as no difference of initial of final body weight was observed. Due to the incomplete genetic correlations between FCR and digestive efficiency, the difference in digestive efficiency between the two groups (17.3%) was lower than the expected 30 to 40% [18,41]. Differences between the two groups for the coefficients of fecal digestive use of starch, lipids and proteins were similar to differences previously observed between the D+ and D- lines [42], the difference being lower for proteins (10.4%) and higher for lipids (37.7%).

Differences between the two FCR groups regarding microbiota composition are shown in Table 2. The groups mainly differed by higher counts for Lactobacillus, *L*. *salivarius* and *E*. *coli* in the FCR_H group (+24.6%, +58.4% and +50.7%, respectively) than in the FCR_L group. These variations in some bacterial groups resulted in differences in equilibrium between bacteria. As *L*. *salivarius* but not *L*. *crispatus* differed between FCR_H and FCR_L, their ratio to Lactobacillus was also different between the two groups, with relatively more *L*. *salivarius* and less *L*. *crispatus* in less efficient birds (P = 0.03 and 0.06, respectively). The ratios of clostridia to lactobacillus and to *E*. *coli* were also higher in efficient than in less efficient birds (P<0.02). The association between the composition of cecal microbiota and feed conversion ratio in chicken has already been reported by Torok et al. [16] and Stanley et al. [11]. In the latter study, contrary to our study, a higher *L*. *crispatus* count was associated with poor efficiency, but their study was based on a very different genotype and diet, which might explain the difference in results.

**Fig 1:**
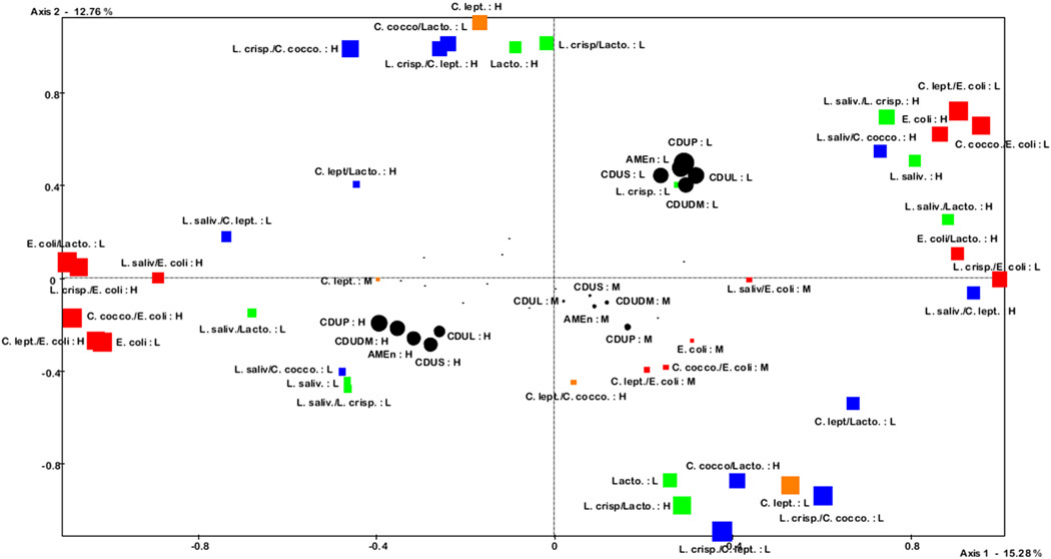
Multiple correspondence analysis of digestive microbiota in cecal contents and digestive efficiency traits. AMEn: metabolisable energy corrected to zero nitrogen retention; CDUDM, CDUL, CDUP, CDUS: coefficients of fecal digestive use of dry matter, lipids, proteins, and starch; Lacto.: Lactobacillus count; L. saliv.: *L*. *salivarius* count in cecal content; L. crisp.: *L*. *crispatus* count in cecal content; C. lept.: *C*. *leptum* count in cecal content; C. cocco.: *C*. *coccoides* count in cecal content; H, M, L: third of individuals with the highest, medium, and lowest values within a trait. The size of the squares is proportional to the relative contribution of the category of active variables to the axes. The size of the circles is proportional to the cos² between the axes and the category of the illustrative variable.

The phenotypic association between microbiota composition and digestive efficiency was seen with the multifactorial analysis (Table 3, Fig 1). It first illustrated that the first factor of variation in microbiota composition was *E*. *coli*, as together *E*. *coli* and the ratio of *E*. *coli* to Lactobacillus, *L*. *crispatus*, *C*. *leptum* and *C*. *coccoides* represented 52.9% of the variability on the first axis. The second axis reflected the equilibrium between Lactobacillus, *L*. *crispatus* and clostridia, with 70.5% of the variability of the axis explained by Lactobacillus, *C*. *leptum*, the ratios of *L*. *crispatus* to Lactobacillus, *C*. *leptum* and *C*. *coccoides* and the ratio of *C*. *coccoides* to Lactobacillus. All traits of digestive efficiency were significantly associated with these two axes, better digestive efficiency being associated with a lower *E*. *coli* count, and thus higher ratios of other bacteria types to *E*. *coli*. On the second axis, better digestive efficiency was also associated with lower Lactobacillus and *C*. *leptum* counts, higher ratios of *C*. *coccoides* and *L*. *crispatus* to Lactobacillus and a lower ratio of *C*. *leptum* to Lactobacillus.

**Table 3:** Results of multiple correspondence analysis between cecal microbiota composition and digestive efficiency.

		Axis	
		1	2
**Eigen Value**		0.3056	0.2552
**Percentage of variance explained by the axis**		15.28	28.04
**Contribution of active variables to MCA analysis (%)**			
**Lactobacillus**		0.63	10.42
***L*. *salivarius***		4.25	2.66
***L*. *crispatus***		1.03	1.91
***C*. *leptum***		2.55	11.81
***C*. *coccoides***		0.89	0.05
***E*. *coli***		9.91	3.16
***L*. *salivarius* / Lactobacillus**		6.05	0.50
***L*. *crispatus* / Lactobacillus**		0.90	12.44
***C*. *leptum* / Lactobacillus**		3.93	3.11
***C*. *coccoides* / Lactobacillus**		1.46	11.06
***E*. *coli* / Lactobacillus**		10.73	0.27
***L*. *salivarius* / *L*. *crispatus***		4.25	4.54
***L*. *salivarius* / *C*. *leptum***		7.13	0.34
***L*. *salivarius* / *C*. *coccoides***		4.04	2.82
***L*. *salivarius* / *E*. *coli***		5.66	0.00
***L*. *crispatus* / *C*. *leptum***		1.15	13.48
***L*. *crispatus* / *C*. *coccoides***		3.05	11.32
***L*. *crispatus* / *E*. *coli***		11.37	0.02
***C*. *leptum* / *C*. *coccoides***		0.08	1.99
***C*. *leptum* / *E*. *coli***		9.99	4.51
***C*. *coccoides* / *E*. *coli***		10.93	3.57
**t-test values for illustrative variables** ^**1**^			
**AMEn** ^**2**^	Low^3^	-2.63	-2.18
	Medium	0.73	-1.00
	High	2.02	3.36
**CDUDM**	Low	-2.94	-1.83
	Medium	0.96	-0.86
	High	2.10	2.84
**CDUL**	Low	-2.18	-1.96
	Medium	0.18	-0.82
	High	2.17	3.00
**CDUS**	Low	-2.28	-2.36
	Medium	0.67	-0.62
	High	1.70	3.14
**CDUP**	Low	-3.28	-1.64
	Medium	1.34	-1.70
	High	2.07	3.52

^1^ a t-test value above 1.96 or below 1.96 means that the position of the category of illustrative variable on the axis is significantly different from zero

^2^ AMEn: metabolisable energy corrected to zero nitrogen balance; CDUDM, CDUS, CDUL, CDUP: coefficients of fecal digestive use of dry matter, starch, lipids and proteins.

^3^ Low, Medium, High: each category includes one third of individual values

These associations between digestive efficiency and microbiota composition are consistent with literature reports. Indeed, it was shown in previous studies that *E*. *coli* and Lactobacillus were more frequent in the chicken ceca when birds were fed a wheat and barley diet, leading to a lower AMEn, than when they were fed a corn diet leading to a higher AMEn [43,44].

The digestive microbiota in the ceca is dependent on the characteristics of this biotope such as the nature and quantity of available substrate and transit time. It has previously been reported that the transit time is much shorter in D- than in D+ birds [45]. This implies that D+ birds have more time to absorb nutrients in the intestine, which promotes the development of bacterial species able to survive in harsh conditions, whereas the development of bacteria able to proliferate fast is favored in D- birds with shorter transit times.

The quantity and nature of nutrients in the ceca indicate that cecal function is more developed in D+ than in D- birds, the former having heavier cecal weights and cecal content than the latter [45]. This may contribute to the better capacity of the FCR_L birds in our study to extract energy and nitrogen from the diet, which thus leads to a higher AMEn and a lower quantity of nitrogen available to be used by bacteria in uric acid fermentation. Moreover, the relationship between cecal digestive microbiota and feed efficiency may be explained in part by the large number of bacteria present in this digestive segment and by their high metabolic capacity to produce volatile fatty acids by fermentation, which provides an additional energy source for birds [46].

The development of cecal bacteria is also influenced by the nature of the substrates that come both from the end of the small intestine and from the retro-peristaltic flux from the cloaca, the latter including urinary products. Undigested compounds from the ileum in our FCR_H and FCR_L groups were probably different as a consequence of their difference in the digestion of proteins, starch and lipids. Moreover, urinary compounds mainly composed of uric acid may also differ between the two groups [47]. Cecal contents may thus be relatively rich in protein and uric acids in FCR_L birds, and conversely relatively rich in starch in FCR_H birds. These different conditions may be responsible for the different bacterial development, for example the positive relationship between digestive efficiency and the *C*. *coccoides* to Lactobacillus ratio might partly be due to the ability of Clostridium spp. to utilize uric acid [48].

On the other hand, the relationship between digestive efficiency and microbiota composition may be due to the effects of microbiota on its host. The cecal digestive microbiota may have an effect on digestive physiology via its production of metabolites such as butyrate, secretion of neuroendocrine hormones and its interactions with the nervous system that innervates the gastrointestinal tract or via neuropeptides [49–52]. Another contribution of cecal microbiota to fecal digestibility is its contribution to the fecal biomass. However, in the case of the D+ and D- lines, this contribution is probably minor as D+ animals with the highest fecal digestibility appeared to have more developed cecal microbiota [2].

### Heritability

Heritability of microbiota composition reflects the proportion of this composition that can be attributed to the genetics of the host. As they were calculated on a low number of animals, the genetic parameters presented in Table 4 have to be taken as indicative values. Heritability estimates were generally low. However, among the bacterial groups or ratios that differed between FCR_H and FCR_L birds, the ratios of *L*. *crispatus*, *C*. *leptum* and *C*. *coccoides* to *E*. *coli* presented significant heritability estimates (between 0.16 and 0.24). *E*. *coli* and the ratio of *E*. *coli* to Lactobacillus presented similar heritability estimates but, due to higher standard error estimates, they were not significant. By comparing the composition of fecal microbiota in lines selected for high or low body weight, Zhao et al. [4] and Meng et al. [10] also found that microbiota differed between the two lines and that several species presented heritability. As in our study, they found that *L*. *salivarius* was not heritable.

**Table 4:** Heritability (± standard errors) of microbiota composition and genetic correlations (± standard errors) between microbiota composition and digestive efficiency.

Trait	h²	Genetic correlations with				
		AMEn^1^	CDUDM	CDUS	CDUL	CDUP
**Log(*L*. *salivarius***)	0.048 ± 0.071	-0.084 ± 0.526	-0.333 ± 0.409	0.095 ± 0.639	-0.543 ± 0.572	**-0.672 ± 0.269**
**Log(*L*. *crispatus***)	0.080 ± 0.058	0.235 ± 0.589	**0.764 ± 0.388**	-0.108 ± 0.623	0.230 ± 0.593	-0.255 ± 0.617
**Log(Lactobacillus**)	0.067 ± 0.069	-0.428 ± 0.355	0.043 ± 0.413	**-0.599 ± 0.275**	-0.553 ± 0.453	**-0.850 ± 0.235**
**Log(*C*. *leptum***)	0.155 ± 0.134	0.212 ± 0.156	**0.508 ± 0.184**	-0.377 ± 0.233	-0.152 ± 0.197	-0.035 ± 0.452
**Log(*C*. *coccoides***)	0.037 ± 0.082	**0.476 ± 0.176**	**0.503 ± 0.206**	0.267 ± 0.226	-0.063 ± 0.347	-0.278 ± 0.850
**Log(*E*. *coli***)	0.164 ± 0.109	-0.175 ± 0.207	**-0.658 ± 0.252**	-0.027 ± 0.310	**-0.642 ± 0.289**	-0.061 ± 0.614
**Log(*L*. *salivarius***)**/ Log(*L*. *crispatus***)	0.150 ± 0.159	-0.221 ± 1.545	-0.710 ± 0.965	0.080 ± 1.575	-0.605 ± 1.435	-0.408 ± 0.669
**Log(*L*. *salivarius***)**/ Log(Lactobacillus**)	0.067 ± 0.056	0.242 ± 0.304	-0.337 ± 0.324	0.531 ± 0.368	-0.111 ± 0.442	0.041 ± 0.764
**Log(*L*. *salivarius***)**/ Log(*C*. *leptum***)	0.080 ± 0.100	-0.095 ± 0.531	-0.391 ± 0.499	0.307 ± 0.601	-0.285 ± 0.671	-0.579 ± 0.319
**Log(*L*. *salivarius***)**/ Log(*C*. *coccoides***)	0.019 ± 0.044	-0.180 ± 0.741	-0.391 ± 0.767	0.075 ± 0.835	-0.501 ± 0.799	-0.736 ± 0.391
**Log(*L*. *salivarius***)**/ Log(*E*. *coli***)	0.057 ± 0.065	0.202 ± 0.602	0.691 ± 0.414	0.067 ± 0.664	0.505 ± 0.604	-0.302 ± 0.738
**Log(*L*. *crispatus***)**/ Log(Lactobacillus**)	0.179 ± 0.108	0.651 ± 0.389	**0.958 ± 0.151**	0.340 ± 0.538	**0.780 ± 0.323**	0.483 ± 0.469
**Log(*L*. *crispatus***)**/ Log(*C*. *leptum***)	0.077 ± 0.049	0.192 ± 0.663	0.756 ± 0.428	-0.036 ± 0.688	0.342 ± 0.616	-0.278 ± 0.599
**Log(*L*. *crispatus***)**/ Log(*C*. *coccoides***)	**0.136 ± 0.077**	0.233 ± 0.499	**0.803 ± 0.297**	-0.064 ± 0.575	0.410 ± 0.479	-0.044 ± 0.570
**Log(*L*. *crispatus***)**/ Log(*E*. *coli***)	**0.235 ± 0.094**	0.102 ± 0.378	**0.694 ± 0.295**	-0.111 ± 0.425	0.522 ± 0.481	-0.044 ± 0.681
**Log(*C*. *leptum***)**/ Log(Lactobacillus**)	0.086 ± 0.065	0.575 ± 0.311	**0.274 ± 0.292**	0.367 ± 0.274	0.494 ± 0.431	**0.960 ± 0.282**
**Log(*C*. *leptum***)**/ Log(*C*. *coccoides***)	0.126 ± 0.136	-0.346 ± 0.611	0.069 ± 0.525	-0.754 ± 0.477	-0.213 ± 0.715	-0.053 ± 0.568
**Log(*C*. *leptum***)**/ Log(*E*. *coli***)	**0.207 ± 0.055**	0.168 ± 0.238	**0.702 ± 0.226**	-0.172 ± 0.284	**0.528 ± 0.257**	0.043 ± 0.520
**Log(*C*. *coccoides***)**/ Log(Lactobacillus**)	**0.102 ± 0.058**	**0.638 ± 0.151**	0.116 ± 0.184	0.758 ± 0.392	0.559 ± 0.460	0.749 ± 0.523
**Log(*C*. *coccoides***)**/Log(*E*. *coli***)	**0.156 ± 0.076**	0.294 ± 0.369	**0.765 ± 0.271**	0.045 ± 0.403	**0.674 ± 0.302**	0.142 ± 0.352
**Log(*E*. *coli***)**/ Log(Lactobacillus**)	0.208 ± 0.135	0.021 ± 0.461	-0.597 ± 0.399	0.223 ± 0.486	-0.380 ± 0.426	0.258 ± 0.597

Bold values are significantly different from 0 (P<0.05)

^1^ AMEn: metabolisable energy corrected to zero nitrogen retention; CDUDM, CDUS, CDUL, CDUP: coefficients of fecal digestive use of dry matter, starch, lipids and proteins

The presence of significant heritability for some microbiota components and of genetic correlations between microbiota and digestive efficiency is consistent with the expected effects of host genetics on microbiota. By selecting more and less efficient birds, we first changed the quantity of undigested nutrients in the ceca that are used as growth substrates by bacteria. Moreover, several earlier studies on these lines showed that the biotope had been considerably modified in the small intestine, which also affected the conditions of microbiota development. Indeed, we showed that acid secretions and bile acid secretions [17], gut motility [45] and the structure of the intestinal epithelium [18,53] were very different between the two lines. All these parameters may explain an indirect effect of host genotype on microbiota through modification of the microbiota biotope [4]. Finally, the presence of several QTLs for digestive and feed efficiency which are located on GGA16, that also carries the major histocompatibility complex, highlights the fact that the birds’ immune system had probably been affected during the selection process, which would also directly affect the relationship between host and bacteria.

Both positive and negative genetic correlations were found between digestive efficiency and microbiota composition. As for phenotypic correlations, we found negative genetic correlations between Lactobacillus and starch and protein digestibility, and between *E*. *coli* and digestibility of dry matter and lipids. The negative correlation between Lactobacillus and starch digestibility may be due to the fact that the ceca of FCR_H group contain more undigested starch that can be used by lactobacillus in fermentation in the ceca, thus favoring its development [54]. Similarly, the negative correlation between digestibility of lipids and *E*. *coli*, *L*. *crispatus/E*. *coli* or *C*. *coccoides/E*. *coli* may be due to the negative effect of *E*. *coli* on lipid digestibility in the small intestine. A greater amount of *E*. *coli* decreases the digestibility of lipids and thus increases the quantity of undigested lipids in the ceca, that can in turn be used by *E*. *coli* for its growth in this segment [55,56]. This negative relationship between fecal lipid digestibility and the presence of *E*. *coli* in the ceca has already been reported by Rodriguez et al. [43]. The negative correlation may also originate from the effects of bacteria on the host, through the production of unfavorable metabolites, their secretion of neuroendocrine hormones and their interactions with the enteric nervous system [52].

However, not all relationships between microbiota and digestive efficiency were negative. Indeed, several positive correlations were observed between AMEn and *C*. *coccoides* and the *C*. *coccoides* to Lactobacillus ratio. This may be explained by the caloric extraction from undigestible polysaccharides by this Clostridium spp. which may increase energy available to the host [6]. The positive genetic correlations between protein digestibility and the ratio of *C*. *leptum* to lactobacillus (and *C*. *coccoides* to Lactobacillus although not significant) could be explained by the ability of Clostridium spp. to metabolize amino acids [57] and to degrade uric acid into ammonia that can be used by the host to synthesize amino acids [58]. Clostridium spp. and Lactobacillus may also be involved in regulation of the digestive physiology via the production of beneficial active biological compounds [59,60].

### QTL for microbiota

We detected a total of 14 QTLs that influence the composition of the microbiota (Table 5). They were all significant only on the chromosome-wide scale. However, the QTL for *C*. *leptum* count on chromosome 6, which was significant in all six sire families, was quite close to the genome-wide significance level (P = 0.09). For 10 of these 14 QTLs, *C*. *leptum* and lactobacillus were involved. This higher frequency of QTL in these two groups is consistent with their frequency in cecal microbiota in this study [61]. Moreover, Clostridium spp. are able to degrade the non-starch polysaccharides of wheat [62,63], and it is thus possible that our selection on digestive efficiency with Rialto wheat specifically affected these bacteria. The effects of these QTLs were quite high, which is partly due to the fact that we used birds with extreme FCR values for detection.

**Table 5:** QTL detected for microbiota composition.

QTL number	Trait	GGA	Position (M)	Confidence interval (M)^1^	Markers flanking the confidence interval	NF^2^	Effect^4^	Chromosomewide P level	Genome wide P level	N genes in the region^6^
1	Log(*L*. *crispatus*)/ Log(*C*. *coccoides*)	1	2.560	2.541–2.567	Gga_rs13911828, GGa_rs13913250	3F^3^	0.814	0.048	NS^5^	14
2	Log(*C*. *leptum*)/ Log(Lactobacillus)	2	2.390	2.325–2.400	Gga_rs15142674, GgaluGA164535	5	0.552	0.047	NS	42
3	Log(*L*. *salivarius*)/ Log(Lactobacillus)	3	2.320	2.301–2.336	Gga_rs16324984, Gga_rs14399484	2	0.860	0.037	NS	18
4	Log(*L*. *crispatus*)/ Log(*C*. *leptum*)	6	0.630	0.581–0.839	Gga_rs14579919, Gga_rs15807987	3	0.677	0.028	NS	220
5	Log(*C*. *leptum*)	6	0.920	0.912–0.950	Gga_rs15813564, Gga_rs16565135	6	0.895	<0.0001	0.090	36
6	Log(*C*. *leptum*)/ Log(Lactobacillus)	6	0.980	0.620–1.009	GgaluGA300856, Gga_rs16006607	5	0.903	0.043	NS	306
7	Log(*C*. *leptum*)/ Log(Lactobacillus)	8	0.600	0.000–0.141	Gga_rs15892308, GgaluGA323478	4F	0.605	0.023	NS	129
8	Log(Coli)/ Log(Lactobacillus)	12	0.090	0.081–0.115	Gga_rs15632811, Gga_rs14032854	4F	0.520	0.029	NS	50
9	Log(*L*. *crispatus*)/ Log(*C*. *coccoides*)	14	0.240	0.235–0.246	GgaluGA101400, GgaluGA101629	3	0.897	0.029	NS	8
10	Log(*L*. *crispatus*)	14	0.240	0.235–0.244	GgaluGA101400, Gga_rs14074053	2F	1.241	0.019	NS	6
11	Log(*L*. *salivarius*)/ Log(Lactobacillus)	14	0.480	0.440–0.561	Gga_rs15738570, GgaluGA104485	3	0.591	0.014	NS	64
12	Log(*C*. *leptum*)/ Log(*C*. *coccoides*)	18	0.153	0.143–0.324	GgaluGA119123, GgaluGA121355	4	0.476	0.048	NS	152
13	Log(*L*. *salivarius*)/ Log(*C*. *leptum*)	21	0.480	0.429–0.520	Gga_rs14285137, Gga_rs14286198	3F	0.605	0.045	NS	53
14	Log(*L*. *salivarius*)/ Log(Lactobacillus)	26	0.310	0.268–0.344	GgaluGA196721, Gga_rs15235289	3	0.532	0.048	NS	74

^1^ 1-LOD-drop off confidence interval (lower and upper boundaries, cM)

^2^ Number of F1 sires families heterozygous for the QTL (P<0.05, Student test)

^3^ F: the QTL is fixed in F1 sires families in which it is significant

^4^ QTL effect as a proportion of the phenotypic standard deviation of trait

^5^ NS: P>0.150 at the genome wide level

^6^ Number of genes in the QTL region, as found by http://annotqtl.genouest.org/ interrogation

Several of these QTLs co-localized with QTLs for other traits detected in the same experiment. First, co-localizations were found with the anatomy of the gut, such as intestine and particularly ileum weight and length (with QTLs 2, 7, 13 and 14 [17,64]) and proventriculus weight (with QTLs 4 and 6, [17]). Secondly, QTLs 6 and 8 co-localized with the QTL for breast yield [64]. Finally, QTLs for *L*. *crispatus* and *L*. *crispatus* to *C*. *coccoides* ratio co-localized with a QTL for feeding behavior (unpublished data).

The QTL for *C*. *leptum* count on chromosome 6 was close to genome-wide significance, and we therefore looked for potential candidate genes in this region. Relevant genes present in this region are mainly linked to the inflammatory response of the intestine, which is consistent with previous results in mammals showing the contribution of host genetics to the digestive microbiota community [15]. Indeed, most of the genes shown to have an impact on the composition of gut microbiota are components of the immune system. In this study, candidate genes appeared in the Toll-Like Receptor (TLR) and the transforming growth factor β (TGF-β) pathways. This is consistent with the role of intestinal bacteria in the stimulation of immune system development and induction of a continuous anti-inflammatory response in the host [61,65].

The TLR receptors present in the intestinal epithelium are able to recognize molecular patterns present in microbial cell walls and are the first line of defense in the immune response of the host to microbes. They have also been shown to be involved in the control of microbiota in mice [66]. They activate transcription factor NFκB, that in turn regulates the expression of genes of both innate and adaptive immunity, including inflammatory cytokines [67,68]. Most commensal bacteria, including *C*. *leptum*, are able to limit the immuno-regulatory NFkB pathway by their production of butyrate [6,69]. The genes linked to NFκB in our QTL zone were i) DMTB1 which activates NFκB and has been shown to limit intracellular invasion by *Salmonella enterica* [70], ii) C6H10ORF46, the neddylation of which can be reduced by bacteria, which in turn blocks the NFκB pathway [69], iii) GRK5 the expression of which inhibits the expression of NFκB [68], and iv) PRDX3 which interacts with MAP3KIA to regulate expression of NFκB [71].

TGF-β is a cytokine involved in the secretion of interleukin 17 (IL-17) by Th17 and Treg cells which contribute to the inflammatory process in the intestine [72], and its production is stimulated by the presence of *C*. *leptum* [6,73]. Within the TGF-β pathway, our QTL zone contained i) HTRA1 which inhibits signals from TGF-β family members [74], ii) RAB11FIP2 which is involved in the limitation of inflammatory responses to commensal bacteria through its action of compartmentalization of Toll-Like receptors [75], iii) ADAM12 which limits the production of IL-17 by Th17 cells [72], and iv) C10ORF88 which is also involved in the secretion of IL-17 by Th17 cells through its effect on vitamin D metabolism [76]. Vitamin D also regulates the innate immune response to microbiota [77].

In addition, the PSTK gene within the QTL for *C*. *leptum* (GGA6), which contributes to the selenocysteine secretion pathway, is used by bacteria to fix selenium, a trace element that has an anti-apoptotic function in the colonic crypts and contributes to the integrity of the intestinal mucosa [78]. This gene is also involved in the regulation of calcium metabolism which is required for normal early muscle development, which is also consistent with the fact that this QTL co-localizes with a QTL for breast yield found in the same experiment [64].

Finally, our QTL region for *C*. *leptum* on GGA6 also includes genes involved in the regulation of intestinal motility such as VMAT2 and BAG3, which to our knowledge has not been reported before. BAG3 is expressed in the muscular layer of the intestine and has a probable function in the regulation of motility of the intestine [79]. VMAT2 is a transporter of catecholamines such as dopamine and norepinephrine that affect both immunity and motility of the intestine and have been shown to be stimulated by the presence of Clostridium spp., including *C*. *leptum* [6]. This is also consistent with our previous results on the D+ and D- lines showing wide differences in transit times between the two lines, particularly in the ceca [45], and this factor may be involved in modification of digestive microbiota (including clostridia), as has been reported in the mouse [80].

## Conclusion

Our study clearly demonstrates the existence of genetic control by the host of its microbiota, and the link between host genetics, microbiota composition and feed and digestive efficiency. Lactobacillus, *C*. *leptum* and *E*. *coli* were identified as the most important factors in this interaction. Moreover, the equilibrium between the different categories of bacteria is also an important element of this interaction. The QTL for *C*. *leptum* on chromosome 6 indicates that the inflammatory response of the gut and the motility of the digestive tract are the most probable processes involved. Transcriptomic analyses are underway to confirm the involvement of these candidate genes in determination of microbiota.
